# Supplementary material for: Techno-economic assessment of effervescent tablet-based nanofluids
Source: PLoS One. 2025 Apr 3;20(4):e0319265. doi: 10.1371/journal.pone.0319265 (PMC11967968; doi:10.1371/journal.pone.0319265)
Supplement: S1 Table — (PDF) [file pone.0319265.s001.pdf]

S1 Table. Prices of the equipment, tools, and part used to produce the different nanofluids.

Equipment/tools/part	Nanofluid production route		Cost ($)	Supplier
	Conventional two-step	Effervescent tablet		
Dry powder mixing device	–	X	4,000	Foshan Xingle Machinery Equipment
Glove box	X	X	26,000	MBRAUN
Probe type Sonicator	X	–	~7,946	Fisher Scientific
Hot/cold plate	X	–	1,200	Thermtest Instruments
Analytical Balance	X	X	~699	US Solid
Pneumatic compression instrument	–	X	~596	SHIMADZU Corporation
Tablet die (25 mm)	–	X	438	MTI Corporation
Magnetic Stirrer	X	–	~160	ONiLAB
Agate mortar and pestle	–	X	~120	Deschem
Glass vials	X	–	65.832	Sigma-Aldrich® Solutions
Magnetic stir bars	X	–	5.069	Sigma-Aldrich® Solutions
Sealing tablets bag	–	X	0.36	YURUI Factory
