# Supplementary material for: Techno-economic assessment of effervescent tablet-based nanofluids
Source: PLoS One. 2025 Apr 3;20(4):e0319265. doi: 10.1371/journal.pone.0319265 (PMC11967968; doi:10.1371/journal.pone.0319265)
Supplement: S2 Table — (PDF) [file pone.0319265.s002.pdf]

S2 Table. Prices of powders used to produce the different nanofluids.

**Item**	**Quantity (mg)**	**Nanofluid production approach**		**Cost ($/g)**	**Supplier**
		**Conventional two-step**	**Effervescent tablet**		
MWCNTs	~3,366	X	X	5.2	SkySpring Nanomaterials
SDS	~3,366	X	X	3.1	Sigma-Aldrich® Solutions
NaH_2PO_4	~68,673	–	X	10.1	Sigma-Aldrich® Solutions
Na_2CO_3	~20,198	–	X	0.7	Sigma-Aldrich® Solutions
