# Supplementary material for: Techno-economic assessment of effervescent tablet-based nanofluids
Source: PLoS One. 2025 Apr 3;20(4):e0319265. doi: 10.1371/journal.pone.0319265 (PMC11967968; doi:10.1371/journal.pone.0319265)
Supplement: S3 Table — (PDF) [file pone.0319265.s003.pdf]

S3 Table. Amount of electricity required to run the devices and its cost.

Device/equipment	Power (W)	Operating time for single product (hour)	Electrical consumption (Wh)	Consumed electricity cost (S)		
				LEC	AEC	HEC
Glove box	4,500	0.50	2,250	1.58E-02	3.71E-01	1.56
Dry powder mixing device	1,500	0.33	500	3.50E-03	8.25E-02	3.46E-01
Probe type Sonicator	750	0.20	150	1.05E-03	2.48E-02	1.04E-01
Magnetic Stirrer	200	0.08	17	1.19E-04	2.81E-03	1.18E-02
Hot/cold plate	100	0.20	20	1.40E-04	3.30E-03	1.38E-02
Analytical Balance	12.5	0.75	9.4	6.58E-05	1.55E-03	6.50E-03
