# Supplementary material for: Techno-economic assessment of effervescent tablet-based nanofluids
Source: PLoS One. 2025 Apr 3;20(4):e0319265. doi: 10.1371/journal.pone.0319265 (PMC11967968; doi:10.1371/journal.pone.0319265)
Supplement: S4 Table — (PDF) [file pone.0319265.s004.pdf]

S4 Table. Accumulated cost for the conventional two-step and effervescent tablet-based nanofluid production project at different interest rates.

Production project type	Accumulated cost ($) based on year			Accumulated interest ($) based on year		
	5	10	20	5	10	20
Conventional NF (10% interest)	238,874.3	294,738.9	425,448.9	57,770	113,634.6	244,344.6
Conventional NF (20% interest)	302,788	431,975	743,819	121,683.7	250,870.7	562,714.7
Conventional NF (30% interest)	371,790.4	585,806.2	1,092,373.6	190,686.1	404,701.9	911,269.3
Tablet NF (10% interest)	212,203.2	261,830.3	377,946.1	51,319.8	100,946.9	217,062.7
Tablet NF (20% interest)	268,980.7	383,743.5	660,769.1	108,097.3	222,860.1	499,885.7
Tablet NF (30% interest)	330,278.8	520,399	970,406.4	169,395.4	359,515.6	809,523
