# Supplementary material for: Techno-economic assessment of effervescent tablet-based nanofluids
Source: PLoS One. 2025 Apr 3;20(4):e0319265. doi: 10.1371/journal.pone.0319265 (PMC11967968; doi:10.1371/journal.pone.0319265)
Supplement: S5 Table — (PDF) [file pone.0319265.s005.pdf]

S5 Table. Accumulated interest cost for the conventional two-step and effervescent tablet-based nanofluid production project at different set of interest rates.

Electrical cost category	Total OPEX ($/year) based on project type	
	Conventional	Effervescent tablet
LEC	25,966.99	6,351.87
AEC	26,093.75	6,494.89
HEC	26,516.57	6,971.84
