# Supplementary material for: Techno-economic assessment of effervescent tablet-based nanofluids
Source: PLoS One. 2025 Apr 3;20(4):e0319265. doi: 10.1371/journal.pone.0319265 (PMC11967968; doi:10.1371/journal.pone.0319265)
Supplement: S6 Table — (PDF) [file pone.0319265.s006.pdf]

S6 Table. Changes in total annual cost for the different nanofluid production projects based on the electrical cost and employed interest rate.

Production project type	Total annual cost ($/year) based on employed interest rate			
	One time payment	10% interest	20% interest	30% interest
Conventional NF (LEC)	2,5967	47,239.4	63,157.9	80,585.7
Conventional NF (AEC)	2,6093.8	47,366.2	63,284.7	80,712.4
Conventional NF (HEC)	2,6516.6	47,789	63,707.5	81,135.2
Tablet NF (LEC)	6,351.9	25,249.2	39,390.3	54,872.2
Tablet NF (AEC)	6,494.9	25,392.2	39,533.3	55,015.2
Tablet NF (HEC)	6,971.8	25,869.1	40,010.3	55,492.2
