# Supplementary material for: Techno-economic assessment of effervescent tablet-based nanofluids
Source: PLoS One. 2025 Apr 3;20(4):e0319265. doi: 10.1371/journal.pone.0319265 (PMC11967968; doi:10.1371/journal.pone.0319265)
Supplement: S7 Table — (PDF) [file pone.0319265.s007.pdf]

S7 Table. Nanofluid production cost based on the method of production, electrical cost, and interest rate.

**Production project type**	**Nanofluid production cost ($/L) based on employed interest rate**			
	**One time payment**	**10% interest**	**20% interest**	**30% interest**
Conventional NF (LEC)	51.9	94.5	126.3	161.2
Conventional NF (AEC)	52.2	94.7	126.6	161.4
Conventional NF (HEC)	53	95.6	127.4	162.3
Tablet NF (LEC)	12.7	50.5	78.8	109.7
Tablet NF (AEC)	13	50.8	79.1	110
Tablet NF (HEC)	13.9	51.7	80	111
