# Supplementary material for: Techno-economic assessment of effervescent tablet-based nanofluids
Source: PLoS One. 2025 Apr 3;20(4):e0319265. doi: 10.1371/journal.pone.0319265 (PMC11967968; doi:10.1371/journal.pone.0319265)
Supplement: S8 Table — (PDF) [file pone.0319265.s008.pdf]

S9 Table. Project internal rate of return based on the nanofluid method of production, electrical cost, interest rate, and product quantity per year.

**Production method**	**Interest (%)**	**Internal rate of return (%/Year)**								
		**Case 1**			**Case 2**			**Case 3**		
		**LEC**	**AEC**	**HEC**	**LEC**	**AEC**	**HEC**	**LEC**	**AEC**	**HEC**
Conventional	0	56.6	56.8	57.3	1.2	1.2	1.3	-11.3	-11.3	-11.2
	10	44.9	45.0	45.5	-1.0	-1.0	-0.9	-12.7	-12.7	-12.6
	20	36.0	36.2	36.7	-2.9	-2.9	-2.8	-14.0	-14.0	-13.9
	30	26.2	26.4	26.9	-5.4	-5.4	-5.2	-15.7	-15.7	-15.6
Effervescent tablet	0	76.0	76.1	76.6	4.4	4.4	4.5	-9.4	-9.4	-9.3
	10	64.2	64.4	64.9	2.5	2.5	2.6	-10.5	-10.5	-10.4
	20	55.4	55.6	56.1	1.0	1.0	1.1	-11.4	-11.4	-11.3
	30	45.8	45.9	46.5	-0.8	-0.8	-0.7	-12.6	-12.6	-12.5
