# Supplementary material for: Techno-economic assessment of effervescent tablet-based nanofluids
Source: PLoS One. 2025 Apr 3;20(4):e0319265. doi: 10.1371/journal.pone.0319265 (PMC11967968; doi:10.1371/journal.pone.0319265)
Supplement: S10 Table — (PDF) [file pone.0319265.s010.pdf]

S10 Table. Detailed information on the payback period for conventional and effervescent tablet-based nanofluid projects.

**Production project type**	**Nanofluid sold**			**Electrical cost category**			**Interest rate scheme**				**Project payback period (year)**
	**500 L/year**	**50 L/year**	**10 L/year**	**LEC**	**AEC**	**HEC**	**One time payment**	**10%**	**20%**	**30%**	
Conventional NF	X			X			X				0.2
	X			X				X			0.3
	X			X					X		0.3
	X			X						X	0.4
	X				X		X				0.4
	X				X			X			0.5
	X				X				X		0.6
	X				X					X	0.8
	X					X	X				0.5
	X					X		X			0.7
	X					X			X		0.8
	X					X				X	1.1
		X		X			X				2.1
		X		X				X			2.6
		X		X					X		3.3
		X		X						X	4.4
		X			X		X				3.6
		X			X			X			4.6
		X			X				X		5.7
		X			X					X	7.7
		X				X	X				5.3
		X				X		X			6.6
		X				X			X		8.2

		X				X				X	11.1
			X	X			X				10.4
			X	X				X			13.1
			X	X					X		16.3
			X	X						X	22.2
			X		X		X				18.1
			X		X			X			22.8
			X		X				X		28.3
			X		X					X	38.5
			X			X	X				26.3
			X			X		X			33.1
			X			X			X		41
			X			X				X	55.5
Tablet NF	X			X			X				0.2
	X			X				X			0.2
	X			X					X		0.2
	X			X						X	0.3
	X				X		X				0.3
	X				X			X			0.3
	X				X				X		0.4
	X				X					X	0.5
	X					X	X				0.4
	X					X		X			0.5
	X					X			X		0.5
	X					X				X	0.7
		X		X			X				1.6
		X		X				X			1.8
		X		X					X		2.1

		X		X						X	2.6
		X			X		X				2.7
		X			X			X			3.2
		X			X				X		3.7
		X			X					X	4.5
		X				X	X				3.9
		X				X		X			4.7
		X				X			X		5.4
		X				X				X	6.5
			X	X			X				7.7
			X	X				X			9.2
			X	X					X		10.6
			X	X						X	12.8
			X		X		X				13.5
			X		X			X			16
			X		X				X		18.5
			X		X					X	22.3
			X			X	X				19.7
			X			X		X			23.2
			X			X			X		26.9
			X			X				X	32.4
