# Supplementary figures and images for: Techno-economic assessment of effervescent tablet-based nanofluids
Source: PLoS One. 2025 Apr 3;20(4):e0319265. doi: 10.1371/journal.pone.0319265 (PMC11967968; doi:10.1371/journal.pone.0319265)

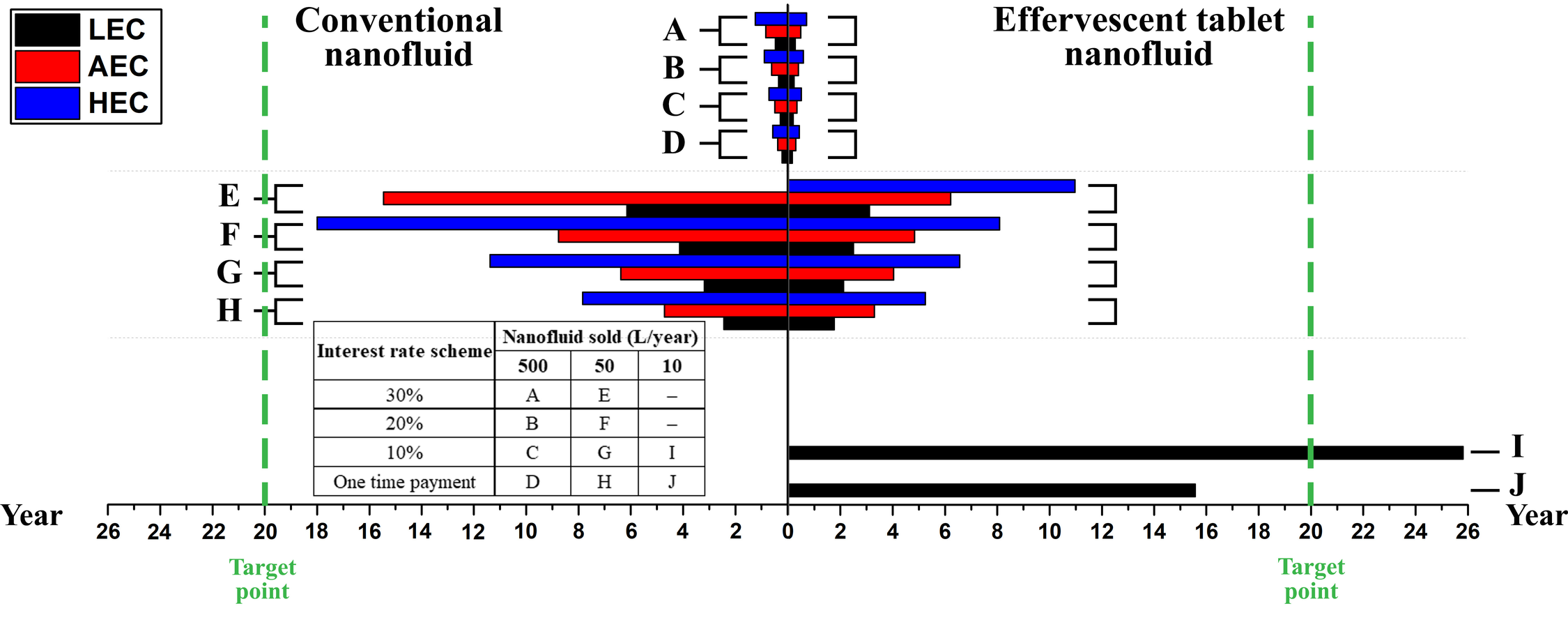

Supplement: S1 Fig — (TIF) [file pone.0319265.s011.tif]
