# Supplementary material for: Techno-economic assessment of effervescent tablet-based nanofluids
Source: PLoS One. 2025 Apr 3;20(4):e0319265. doi: 10.1371/journal.pone.0319265 (PMC11967968; doi:10.1371/journal.pone.0319265)
Supplement: Attachment — Submitted filename: Response to Reviewers.doc [file pone.0319265.s014.doc]

We would like to start by thanking the respected Academic Editor, respected Reviewer 1, and respected Reviewer 2 for taking the time to review our manuscript and for their variable comments and suggestions. Kindly find below our reply on these comments.

**Academic Editor**

**Academic Editor comment:**

1. Please ensure that your manuscript meets PLOS ONE's style requirements, including those for file naming.

**Authors reply:**

We thank the respected Academic Editor for his time and for reviewing our manuscript. Kindly note that the revised version of the manuscript was modified according to PLOS ONE’s style requirements, including those for file naming, as instructed by the respected Academic Editor. Thank you very much.

**Academic Editor comment:**

2. Thank you for stating the following in the Acknowledgments Section of your manuscript:

“The authors acknowledge the financial and technical support that were provided by the funding agency, KFAS (grant no: PN23-35EM-1837), and their institutes, KISR (grant no: EA112C) to complete this research work.”

We note that you have provided funding information that is currently declared in your Funding Statement. However, funding information should not appear in the Acknowledgments section or other areas of your manuscript. We will only publish funding information present in the Funding Statement section of the online submission form.

Please remove any funding-related text from the manuscript and let us know how you would like to update your Funding Statement.

**Authors reply:**

All funding related information was removed from the revised version of the manuscript, as instructed by the respected Academic Editor. We have also written and updated funding statement in our cover latter based on the instructions provided by the respected Academic Editor, which is as following:

The authors acknowledge the financial and technical support that were provided by the Kuwait Foundation for the Advancement of Sciences (KFAS) under project grant no: PN23-35EM-1837, and the Kuwait Institute for Scientific Research (KISR) under project grant no: EA112C to complete this research work. KFAS website: [www.kfas.org](https://linkprotect.cudasvc.com/url?a=https%3A%2F%2Fwww.kfas.org&c=E,1,cPRU2BPTCxFIf2aAEWkunalVjJZUWQIXXOulUvqPN0D0JFtlYfrpucdiLYM5jyp9Y-7fMaKjPae_-WyS4sNBg3MEY4quaaVUi6CNIq6zwaQd1jotz97J_g,,&typo=1&ancr_add=1); and KISR website: [www.kisr.edu.kw](http://www.kisr.edu.kw/)

Thank you very much.

**Academic Editor comment:**

3. We note that Figures 1 and 2 in your submission contain copyrighted images. All PLOS content is published under the Creative Commons Attribution License (CC BY 4.0), which means that the manuscript, images, and Supporting Information files will be freely available online, and any third party is permitted to access, download, copy, distribute, and use these materials in any way, even commercially, with proper attribution. For more information, see our copyright guidelines: http://journals.plos.org/plosone/s/licenses-and-copyright.

We require you to either (1) present written permission from the copyright holder to publish these figures specifically under the CC BY 4.0 license, or (2) remove the figures from your submission

**Authors reply:**

We have replaced Figures 1 and 2 with new figures in our modified version of the manuscript. Please see a comparison between the figures that were referred to in the literature (<https://doi.org/10.1016/j.molliq.2023.123083>) with the new ones that were included in the modified version of the manuscript below. Thank you very much.

| **Figure no.** | **Literature** | **In revised manuscript** |
| --- | --- | --- |
| 1 |  |  |
| 2 |  |  |

**Academic Editor comment:**

4. Please remove your figures from within your manuscript file, leaving only the individual TIFF/EPS image files, uploaded separately. These will be automatically included in the reviewers’ PDF.

**Authors reply:**

All figures have been removed from the revised version of the manuscript as instructed by the respected Academic Editor. Thank you very much.

**Academic Editor comment:**

5. Please review your reference list to ensure that it is complete and correct. If you have cited papers that have been retracted, please include the rationale for doing so in the manuscript text, or remove these references and replace them with relevant current references. Any changes to the reference list should be mentioned in the rebuttal letter that accompanies your revised manuscript. If you need to cite a retracted article, indicate the article’s retracted status in the References list and also include a citation and full reference for the retraction notice.

**Authors reply:**

The reference list has been revised and modified in the revised version of the manuscript as instructed by the respected Academic Editor. Thank you very much.

**Reviewer 1**

**Reviewer 1 comment:**

The novelty of this paper is evident, but it is unfortunately difficult to read due to numerous grammatical and syntactical errors. Some sentences are overly complex and require simplification for clarity and conciseness.

**Authors reply:**

We thank the respected reviewer for reviewing our manuscript and for pointing this out. Kindly note that the revised version of the manuscript was sent to a language editing service to improve its readability and revise all grammatical and syntactical errors. Accordingly, all errors were fixed along with the text flow. Kindly check below the language editing certificate. We do apologies for the previous errors included in our initial manuscript. Thank you very much

**Reviewer 1 comment:**

Additionally, the introduction covers several topics—energy demand, nanotechnology, nanofluids, and methods of fabrication—without a clear central theme. It is also excessively lengthy and repetitive.

**Authors reply:**

We thank the respected reviewer for the provided comment, which we agree with and were happy to take into account. Kindly note that the Introduction Section was revised to focus on nanofluids. As such, we have modified and shortened it by removing unnecessarily parts. Kindly check Lines 53-123. Thank you very much.

**Reviewer 1 comment:**

In the research methods section, the correspondence between terms and variables in the equations is unclear.

**Authors reply:**

We thank the respected reviewer for pointing this out. Kindly note that we have modified our revised version to further clarify the correspondence between terms and variables in the equations, as suggested by the respected reviewer. Kindly check the Research Methodology Sections 2.1, 2.2, 2.3, 2.4, and 2.5. All changes and modifications are in red text. Thank you very much.

**Reviewer 1 comment:**

Figure 1 appears in both this document and the article “Effervescent Tablets for Carbon-Based Nanofluids Production” on ScienceDirect. While it is cited, it is not clear that the figure is sourced from that paper. The same issue applies to Figure 2.

**Authors reply:**

The authors have changed both figures in their revised version of the manuscript. Kindly see below a comparison between our two figures and the ones in the provided literature. Thank you very much.

| **Figure no.** | **Literature** | **In revised manuscript** |
| --- | --- | --- |
| 1 |  |  |
| 2 |  |  |

**Reviewer 1 comment:**

Equations 12-14 could be improved by adding more acronyms to enhance readability and conciseness.

**Authors reply:**

The authors thank the respected reviewer for the suggestion, which we were happy to take into account in our revised version of the manuscript. Kindly note that more acronyms were included in the pointed-out equations. Kindly check Sections 2.4 and 2.5. Thank you very much.

**Reviewer 1 comment:**

In Figures 11 and 12, the legend includes identifiers that do not match those in the graphs. Additionally, Figure 14 is difficult to read.

**Authors reply:**

The authors apologies for the poor illustration. We have changed the illustration of the pointed-out figures to better present the data and eliminate the over lapping. Kindly check new figures 10-13. In addition, please note that the Fig. numbers has changed because we have moved one of the manuscript figures to the Supplementary Information Section. Thank you very much.

**Reviewer 1 comment:**

In the conclusion, the presentation of information and data needs to be clearer and more concise.

**Authors reply:**

The authors thank the respected reviewer for the comment. Kindly note that the Conclusion Section was modified to have a clearer presentation of the information, as suggested by the respected reviewer. Thank you very much.

**Reviewer 2**

**Reviewer 2 comment:**

On page 18, replace the statement ‘For instant…’ with ‘For instance,’ the author should review most of the result and discussion section for any grammatical error.

**Authors reply:**

We thank the respected reviewer for reviewing our manuscript and for pointing this out. Kindly note that the revised version of the manuscript was sent to a language editing service to improve its readability and revise all grammatical and syntactical errors. Accordingly, all grammatical errors were fixed along with the text flow. Kindly check below the language editing certificate. Thank you very much and we do apologies for the previous errors included in our initial manuscript.

**Reviewer 2 comment:**

Statistically speaking, Fig. 6 shows no substantial differences in total OPEX values among the scenarios examined. This raises the question of whether the different nanofluid production methods, based on electrical costs, have a minimal effect on OPEX for both tablet and conventional production. The authors may want to provide further clarification, given the narrow range of values.

**Authors reply:**

We thank the respected reviewer for his comment. Kindly note that the following statement was added to the revised version of the manuscript (Lines 385-388) for further clarification:

‘Notably, the electrical cost represents 0.02% (LEC) to 2.09% (HEC) and 0.10% (LEC) to 8.98% (HEC) of the total yearly OPEX cost for the conventional and effervescent tablet production methods, respectively. Further information on the values used to generate Fig 6 is presented in S5 Table.’

Thank you very much.

**Reviewer 2 comment:**

Would it be possible to tabulate the main economic indicators/metrics or values from sections 3.2 to 3.4 for easier comprehension? The current display of data is extensive and may obscure the key points.

**Authors reply:**

We thank the respected reviewer for his comment. Kindly note that we have tabulated the data from sections 3.2 to 3.4, as suggested by the respected reviewer. Kindly check S4 Table, S5 Table, S6 Table, and S7 Table in the Supplementary Information Section. Thank you very much.

**Reviewer 2 comment:**

Fig. 9 and 10, seem to be quite a complex figure with many details that may throw the reader off. If the authors can create a table for the keys/legend, that clearly describes each alphabet from A to L. If the authors can generate a table that simplifies the results from the plot, it would be clear to the readers.

**Authors reply:**

We thank the respected reviewer for his comment. Kindly note that we have included a table in the two pointed-out figures, as suggested by the respected reviewer. Kindly check Fig 9 and S8 Fig. Also, please check S10 Table and S11 Table. Thank you very much.

**Reviewer 2 comment:**

A lot of graphs/plots and tables shown can be moved to the supplementary information (SI). For example, Table 6 can be sent to SI.

**Authors reply:**

As suggested by the respected reviewer, we have removed previously Table 6 (new S9 Table) to the SI along with other Tables and Figure. Kindly check in the SI Section: S1 Table, S2 Table, S3 Table, and S8 Fig. Thank you very much.

**Reviewer 2 comment:**

From the TEA study, the authors proposed that the tablet-based nanofluid is more economical compared to the conventional approach. Is it possible for the authors to comment on the minimum selling price of these prepared nanofluids to support the production cost?

**Authors reply:**

The authors thank the respected reviewer for his comment. Kindly note that we have commented on the minimum selling price of these prepared nanofluids to support the production cost in our revised version of the manuscript, as following:

‘However, this can be overcome by adjusting the selling price of the product to support its production costs. This is achieved by setting the in Eq. 16 to zero and thus determine the appropriate nanofluid selling price for each TAC, interest rate, and OPEX.’. Thank you very much.

Finally, we would like to thank the respected Academic Editor, respected Reviewer 1, and respected Reviewer 2 for their time, comments, and suggestions, which helped in improving our manuscript.

Thank you all very much.
