# Supplementary material for: Techno-economic assessment of effervescent tablet-based nanofluids
Source: PLoS One. 2025 Apr 3;20(4):e0319265. doi: 10.1371/journal.pone.0319265 (PMC11967968; doi:10.1371/journal.pone.0319265)
Supplement: Attachment — Submitted filename: Response_to_Reviewers_auresp_2.doc [file pone.0319265.s015.doc]

We would like to start by thanking the respected Academic Editor, respected Reviewer 1, and respected Reviewer 2 for taking the time to review our manuscript and for their variable comments and suggestions. Kindly find below our reply on these comments. Please note that all changes are **highlighted in yellow** in the revised version of the manuscript.

**Reviewer 1**

**Reviewer 1 comment:**

The authors did a great job with the edits! However, there are still a few issues in the paper. Firstly, the figures are not displaying correctly in the manuscript.

**Authors reply:**

We thank the respected reviewer for reviewing our manuscript and for pointing this out. Kindly note that the figures were provided to the respected journal in tif format, according to the journal instructions. The pdf builder system does not display them in the manuscript in their full resolution, which is not the case in the final article form. Please find below the link to the figures in their full resolution, which will appear in the published version of the article, if accepted.

<https://www.dropbox.com/scl/fo/1n8lk26960px0iaq4u4u6/ALzvjqlDnX9AGETxHbRjbcQ?rlkey=wwya649ohowi0ydhdnlvs4fls&st=f8hpgn7l&dl=0>

Thank you very much.

**Reviewer 1 comment:**

Additionally, I prefer not to have five equations presented in a row. The authors might consider including and explaining the variables in Equations 1 and 2 before presenting Equations 3 to 5, which could enhance clarity.

**Authors reply:**

We thank the respected reviewer for the provided comment, which we agree with and were happy to take into account. Kindly check Pages 8-9. Thank you very much.

**Reviewer 1 comment:**

I would also appreciate a citation of at least one previous study that utilizes fixed pricing models based on production costs.

**Authors reply:**

We thank the respected reviewer for pointing this out. Kindly note that the following reference was included as part of our citations in the revised version of the manuscript:

Doi: 10.1016/j.enconman.2024.118110

Kindly check reference 41 in Page 12, and in the reference list section. Thank you very much.

**Reviewer 1 comment:**

Furthermore, a table summarizing all the assumptions in the economic model would be helpful.

**Authors reply:**

The authors thank the respected reviewer for their comment, which we were happy to take into consideration in our revised version of the manuscript. Kindly note that a table including a summary of the assumptions used in the economic analysis was included in the revised version of the manuscript. Kindly check Table 3, Page 14.

Thank you very much.

**Reviewer 1 comment:**

The rationale for selecting interest rates from 0% to 30% should be clarified. Are these rates typical for similar projects, or are they hypothetical scenarios?

**Authors reply:**

The authors thank the respected reviewer for pointing this out. Kindly note that the selected interest rates are hypothetical. We have included this in our revised version of the manuscript. Please check Page 11 in the modified version of the manuscript.

Thank you very much.

**Reviewer 1 comment:**

Moreover, the relationship between different rates and the Internal Rate of Return (IRR) should be more explicitly linked to the overall investment strategy.

**Authors reply:**

The authors thank the respected reviewer for his comment. We have tried to address the respected reviewer comment in Section 3.5, Page 25. The following statement was included in the revised version of our manuscript:

In general, the IRR is inversely proportional to the interest rate, showing its lowest value at i = 30% among all three studied cases for both nanofluid fabrication methods. According to Eq. 9 and Eq.16, increasing the interest rate would result in a higher TAC, and consequently leading to a lower net cash flow. The previous causes the IRR value to decrease, according to Eq. 15

Thank you very much.

**Reviewer 1 comment:**

The conclusions could benefit from clearer organization. Presenting them in a list format is less effective than integrating them into a narrative that outlines the key findings and their implications. A more logical flow would enhance readability.

**Authors reply:**

The authors thank the respected reviewer for the comment. Kindly note that the authors have rewriting the conclusion section to compile with the respected reviewer comment, as best as possible. We hope that the newer version is acceptable by the respected reviewer.

Thank you very much.

**Reviewer 1 comment:**

Overall, these revisions will help strengthen the paper and better convey its contributions to the field.

**Authors reply:**

The authors thank the respected reviewer for the time and comments provided. We hope that we were able to improve the quality of our manuscript and meet the level of acceptance.

Thank you very much.

**Reviewer 2**

**Reviewer 2 comment:**

None required - The authors have addressed the questions and concerns about the manuscript, and the revised version is better.

**Authors reply:**

We thank the respected reviewer for taking the time reviewing our manuscript and for his respected decision.

Thank you very much.
