# Supplementary material for: Techno-economic assessment of effervescent tablet-based nanofluids
Source: PLoS One. 2025 Apr 3;20(4):e0319265. doi: 10.1371/journal.pone.0319265 (PMC11967968; doi:10.1371/journal.pone.0319265)
Supplement: Attachment — Submitted filename: Reviewer Comments.pdf [file pone.0319265.s012.pdf]

The authors attempt the (novel) techno-economic assessment of effervescent tablet-based nanofluids, through the two-step synthesis approach to compare both the conventional and tablet-based nanofluids. The study assessed several financial metrics based on the two case studies, and the result confirmed the superiority of the tablet-based nanofluid over the conventional approach. This is indeed an important study, as these nanofluids are suitable as alternative working fluids in thermal systems, and the authors have done a great detailed study from an economic standpoint. Although in its early stage, tablet-based nanofluids appear to be a viable alternative, owing to their low OPEX, higher thermal performance, and the amenability to work in both laminar and turbulent flow conditions. Indeed, the manuscript provides sufficient details regarding the techno-economic study, and it is favorable to proceed into the next phase. However, the following questions/concerns need to be addressed before the manuscript proceeds to the next phase:

1. On **page 18**, replace the statement 'For instant…' with 'For instance,' the author should review most of the result and discussion section for any grammatical error.
2. Statistically speaking, **Fig. 6** shows no substantial differences in total OPEX values among the scenarios examined. This raises the question of whether the different nanofluid production methods, based on electrical costs, have a minimal effect on OPEX for both tablet and conventional production. The authors may want to provide further clarification, given the narrow range of values.
3. Would it be possible to tabulate the main economic indicators/metrics or values from **sections 3.2 to 3.4** for easier comprehension? The current display of data is extensive and may obscure the key points.
4. **Fig. 9 and 10**, seem to be quite a complex figure with many details that may throw the reader off. If the authors can create a table for the keys/legend, that clearly describes each alphabet from A to L. If the authors can generate a table that simplifies the results from the plot, it would be clear to the readers.
5. A lot of graphs/plots and tables shown can be moved to the supplementary information (SI). For example, **Table 6** can be sent to SI.
6. From the TEA study, the authors proposed that the tablet-based nanofluid is more economical compared to the conventional approach. Is it possible for the authors to comment on the minimum selling price of these prepared nanofluids to support the production cost?
